# Familiarity at Work: Awesome or Contempt? Assessing the Interplay among Familiarity, Leadership and Team Identification

**DOI:** 10.3390/bs13120974

**Published:** 2023-11-26

**Authors:** Laura Petitta, Isabella Lo Castro, Anna Guerriero

**Affiliations:** 1 Department of Psychology, Sapienza University of Rome, Via dei Marsi, 78, 00185 Rome, Italy; 2 Center for Higher Defence Studies, Piazza della Rovere, 83, 00165 Rome, Italy; diafr.usmd.svd.capo@casd.difesa.it; 3 Independent Researcher; anna.guerriero2408@gmail.com

**Keywords:** familiarity at work, group member prototypicality, leadership styles, curvilinear relationship

## Abstract

The purpose of this study was to examine competing hypotheses (positive vs. negative) on how organizational members’ familiarity with multiple stakeholders differentially relates to members’ social identity and perception of leadership styles grounded in relational and emotional factors. Specifically, we developed and tested a conceptual model wherein employees’ familiarity with leaders, colleagues, and externals plays a differential role in predicting the extent to which they identify with their workgroup (i.e., group member prototypicality—GMP) and perceive transformational, authentic, leader–member exchange and servant leadership styles. Moreover, we examined the moderating effect of combat experience. We tested this nomological network using structural equation modeling and invariance analyses on a sample of 435 military personnel from the Italian Army (228 combat, 207 non-combat). Results indicated an invariant pattern of relationships among variables for combat and non-combat sub-samples. Specifically, familiarity with leaders positively predicted all leadership styles and GMP. Familiarity with colleagues positively predicted only GMP, whereas familiarity with externals did not predict GMP or leadership factors. Moreover, post hoc quadratic regressions showed a curvilinear inverted-U-shaped relationship between familiarity with colleagues and GMP. Militaries with low or high levels of familiarity with colleagues reported lower levels of GMP compared to militaries with moderate levels of familiarity with colleagues. Hence, at very high levels of familiarity with colleagues, GMP begins to decrease. Theoretical and practical implications of results are discussed in light of the increasing relevance of relational and emotional factors for military leadership, and the current pandemic and geopolitical turmoil.

## 1. Introduction

Leadership is a core competency of the military [1,2], and requires leaders to influence, plan, coordinate, guide and decide [3] in order to achieve the sobering mission to fight and win. Given the dangerous situations that shape the military [4,5], leadership in the military is explicit, there are no substitutes for leadership (i.e., leaders are needed to influence, plan, coordinate, decide and guide) and followers are indoctrinated into the chain of command and control. However, successful coordination within the military is also grounded in group cohesion and bonding [6], which the literature suggests is strongly associated with members’ social identity [7] or rather, the sense of self from their membership to salient social groups (i.e., group member prototypicality; [8]). Yet, bonding at work, and particularly in the military, also comes with the issue of how close and involved in each other’s personal lives people should be in the workplace (i.e., familiarity at work; [9]). Moreover, military missions increasingly extend beyond conventional warfare and kinetic courses of action [10]. For example, soldiers are now often required to shift roles and render humanitarian assistance [11], protect the population, establish or reinforce political institutions and be involved in infrastructure project management [11]. Therefore, in addition to demonstrating proficiency in conventional warfare, today’s military leaders face the challenges of an effective cultural understanding and communication in order to obtain the active support of the local population to achieve victory [12]. Such conditions highlight not only the core relational aspects of leadership [13] required in military scenarios, but also a critical sociocultural knowledge gap in leader development [14].

**Figure 1:**
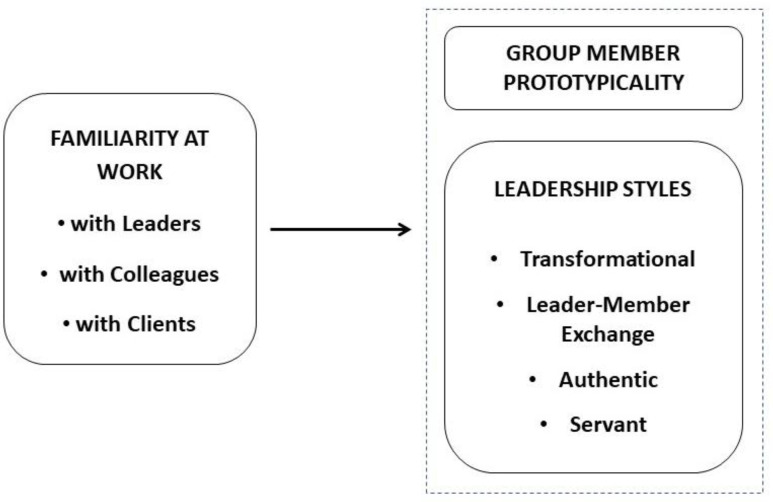
Conceptual model.

The present study responds to this call by examining, in a military context, familiarity at work with leaders, colleagues and externals as a relational antecedent of the social identity of organizational members (i.e., group member prototypicality; GMP) and the leadership styles of their leaders (i.e., transformational, authentic, leader–member exchange, servant) grounded in relational and emotional factors (see Figure 1 for the nomological structure). In line with the interest in relational aspects at work, our research focuses on leadership models that explicitly embed the relational factor in their conceptualization. Moreover, we examine the moderating role of combat vs. non-combat experience of military personnel on the nomological network of the study variables. That is, we test whether our conceptual model linking familiarity, leadership styles and GMP displays similar or different results when comparing soldiers with combat and non-combat experience.

Thus, this study has three main aims, each contributing to the existing literature in a unique way. First, while the literature [11] calls for attention to relationship quality and the understanding of cultural differences in order to shift leadership styles as the situation demands [15], no study has previously investigated the role of familiarity at work with different stakeholders simultaneously. Specifically, we propose an overarching multifaceted conceptualization of familiarity at work, understood as the degree to which an organizational member reports to be familiar with their leader and/or with their colleagues and/or with externals (e.g., civilians, military from other allied nations) based on the feeling to be at ease with and close to others as well as the intentional behaviors of sharing confidential matters and spending time together (e.g., breaks, spare time). It is noteworthy that, in studying familiarity, we target not only an employee’s comfort zone in their relationship with their leader and with colleagues (i.e., organizational members) but also with roles external to the organization. In so doing, we contribute to a better understanding of the complex relational environment that employees, and military personnel in particular, have to face when interacting with other organizational members (i.e., leaders, colleagues) and non-members holding potentially different rules and codes of social interaction.

The second aim of the study was to examine whether and how the familiarity that military personnel experience with multiple stakeholder sources (i.e., leaders, colleagues, and externals), predicts different leadership styles. Specifically, the literature suggests that in addition to tactical skills, leaders require communication and diplomatic skills and the capacity to perceive, manage and employ both social and emotional information to guide reasoning and action [16,17]. As such, leaders are faced not only with the social and emotional demands of combat environments but also with the challenge of understanding and relating to different cultural contexts. Interestingly, the literature stresses that war also requires awareness and management of affect (e.g., grief, joy; [4,18]). Therefore, consistent with the call for attention to the relational and emotional skills of leaders’ actions in the military [5,11], we focus on leadership styles that are manly rooted in (a) relational factors, as is the case of transformational [19], authentic [20] and leader–member exchange [21] leadership models; and (b) emotional factors, as proposed by servant leadership [22]. As such, our study on the link between familiarity (with leaders, colleagues, and externals) and multiple leadership models contributes to expanding the current knowledge on how the relationship that soldiers hold with different organizational roles, and externals in particular (e.g., war zone population, other nations’ military personnel), may impact their perception of the leadership style engaged by their superior.

Finally, we aimed to examine how the familiarity that soldiers experience with multiple stakeholders (i.e., leader, colleagues, and externals) impacts their social identity in the military or rather, the extent to which they identify with their workgroup (i.e., group member prototypicality). Group member prototypicality refers to the individual’s identification with salient social group prototypical characteristics that prescribe members’ behavior, thoughts and feelings in a specific context [23]. The more individuals identify with a group and define the self in terms of the group identity, the more beliefs, emotions and behavior are governed by the group membership (i.e., group member prototypicality; [8]). That is, the stronger the social identity and the more consistent and homogeneous the behaviors of all group members, as is the case of effective military action that requires coordinated action and compliance to leaders’ plans and orders [3]. However, military action increasingly requires effective war skills as well as humanitarian assistance. For example, soldiers’ behaviors that are deemed disrespectful by the local population may impede cooperation and even provoke attacks. Similarly, leaders might be required to secure and stabilize governance of combat environments, thus needing to exert influence not only on their traditional followers, but also on local leaders with whom shared cultural rules and perspectives are lacking. As such, soldiers’ and leaders’ effective action depends on their ability to shift roles with agility (e.g., from combat to assistance) as the situation demands, and on their attention to relationship quality with such “out-group” members [24,25]. Therefore, we argue that the social identity of military personnel is currently facing the challenges of blending the rules and culture norms of one’s own Army (i.e., in-group) with those of people outside the military (i.e., out-group), such as civilians and local populations of foreign countries and/or military from other allied nations.

Overall, the literature on familiarity at work still lacks a systematized conceptualization that includes employees’ experience of familiarity with multiple stakeholders at work (i.e., leaders, colleagues, clients). Moreover, there is scant empirical literature on the relational experience of familiarity in organizations and its effects on leadership action as well as employee social identity that bonds groups in organizations. The present study fills this gap by examining familiarity at work with leaders, colleagues and externals as a relational antecedent of the social identity of organizational members (i.e., group member prototypicality, GMP) and the leadership styles of their leaders (i.e., transformational, authentic, leader–member exchange, servant) rooted in relational and emotional factors (see Figure 1 for the nomological structure).

By examining the familiarity at work (i.e., the feeling of being at ease and sharing time/activities with another person) that militaries diversely hold with their leaders (i.e., those who give them orders), colleagues (i.e., those with whom they collaborate) and externals (i.e., those who they have to protect and/or collaborate with), the current study contributes to a more nuanced understanding of how relational aspects shape leadership action. In so doing, we add to the leadership research by simultaneously testing the role of multiple stakeholders’ foci of familiarity at work, thus bringing informational power to scholars and practitioners on complex and stratified relational dynamics affecting leadership processes in organizations. Moreover, we reconcile the conflicting approaches to workplace bonding/familiarity by testing competing hypotheses (positive vs. negative) on the familiarity–leadership link. Finally, our study contributes to social identity and the GMP literature by testing familiarity at work as a predictor of team member identity (i.e., group member prototypicality) (i.e., GMP). We thereby contribute to bridging the literature on familiarity at work, leadership and social identity.

We begin with a brief overview of the theoretical foundations we draw upon for the different leadership models included in this study. We then present the theoretical background for group member prototypicality and social identity. Next, we define familiarity at work and delineate arguments regarding the relationship between familiarity and leadership as well as familiarity and group member prototypicality. Finally, we test our conceptual model and hypotheses in a field study including 435 militaries with and without combat experience in Italy.

## 2. Theoretical Background

### 2.1. Leadership Models Grounded in Relational and Emotional Factors

Despite the existence of multiple diverse approaches to leadership conceptualization, leadership is commonly defined as the process through which intentional influence on other people is exercised for the purpose of driving, structuring and facilitating activities and relationships within the working group or the organization as a whole [26]. The military leadership literature suggests that strong leadership encourages subordinates to go beyond the obligation to obey and commit to the mission in a way that maximizes their potential, thus emphasizing the centrality of the human element [27]. Moreover, leadership does not occur in a vacuum and the leader–follower–situation triadic relationship is a basic building block of leadership in the military in that all contribute to the outcome of a mission, especially during times of crisis, chaos and complexity when directives might have little effect on exhausted and stressed followers. Interestingly, cohesion and bonding (i.e., relational factors) are not the only major concerns and key ingredients in addressing leadership in the military, but emotion-related factors such as dealing with fear and stress management are also crucial. More importantly, contextual factors and cross-cultural issues are increasingly brought to the forefront. For example, Sweeney and colleagues [5] suggest that leading in the military has the unique feature of dealing with and operating in dangerous contexts and propose a comprehensive and multilevel leadership framework including not only psychological (e.g., social awareness and connection to others) and organizational (e.g., organizational culture) factors, but also more original social and multilayered contextual variables such as the mastery and management of cross-country cultural differences. Indeed, according to Schein [28], organizational culture is a set of norms and values shared among organizational members that the group members have learned to successfully cope with internal and external organizationally relevant problems, and leaders play a crucial role in successfully applying the principles of culture to achieve their organizational goals. Moreover, Hofstede’s [29] cross-cultural framework of cultural dimensions argues that national culture dimensions (e.g., power distance) affect the values of its members and related behaviors, and further contribute to shaping in-group/out-group dynamics.

Similarly, a review of the leadership studies in the U.S. Army [30] suggests that challenges in the military could be successfully addressed by developing and building cross-cutting qualities and skills of all soldiers, and leaders in particular, in the targeted areas of critical thinking (i.e., conceptualized thinking involving the use of reasoning and integration skills, in response to new information or a “new context”, to form a conclusion that will guide one’s behavior, expertise and/or emotion; [31]); resilience (i.e., the ability to persist in the face of difficulties and to bounce back from “adversity”; [32]), adaptability (i.e., an effective change in response to an “altered situation”; [33]); 360° assessment (i.e., well-rounded feedback based on input from peers, supervisors, and subordinates as a milestone “relation-based” mechanism for developing leadership); cross-cultural skills (i.e., increasingly widespread nation-building missions require militaries to interact with individuals from cultures that are vastly different from their own and thus require cross-cultural competencies that allow to forge relationships with the “local populace” in order to successfully accomplish their missions; [30]); and self-development tools (i.e., today’s operating environment makes it difficult for militaries to attend schoolhouse training and has thus emphasized the relevance of taking individual responsibility for self-development, while providing distributed learning methodologies to ensure that development continues during deployments; [30]). Overall, in the military literature, there is burgeoning interest in situational aspects of leadership, particularly in terms of relational and social cross-cultural competencies that extend the context of military action outside the limits of the organizational environment that are becoming the bedrock of military command. As such, the contemporary focus on counterinsurgency and humanitarian operations contributes to shifting the focus on outsider organizational stakeholders such as local populations or militaries from other allied nations (i.e., externals).

In line with the aforementioned interest in relational aspects at work, a recent leadership literature review and systematization [34] proposes a distinction between traditional theories (e.g., transformational leadership, leader–member exchange) and new emerging conceptualizations (e.g., servant leadership, authentic leadership). It is noteworthy that all four approaches are mentioned in major military leadership manuals (e.g., USA, Canadian), even though transformational leadership stands out followed by leader–member exchange, whereas interest in servant leadership is still nascent [5,27]. Moreover, while authentic leadership is less explicitly addressed as it is, its key features of authenticity, ethical standards and trust are actually basic building blocks of a leader’s action and integrity in the military [6]. Briefly, transformational leadership requires the leader to be able to engage and motivate their collaborators in order to pursue common objectives, transforming the surrounding context and, thus, the organization [19,35,36]. A broader model of transformational leadership developed by Podsakoff, MacKenzie, Moorman and Fetter [37] proposes six key dimensions: identifying and articulating a vision, referring to the conduct of the leader aimed at identifying new opportunities for the group or organization, identifying goals for the future and inspiring others with their dreams; providing an appropriate model, related to behavior and example that the leader promotes in order to provide a broad consensus of values to their employees; fostering acceptance of group goals, related to leader behaviors tending towards cooperation among employees for the achievement of common goals; high performance expectations, referring to behavior demonstrations in which the leader expects high work quality and excellent performance from his/her employees; providing individualized support, referring to the leader’s conduct in demonstrating respect for his employees and relevant interest in their needs and feelings; and intellectual stimulation, referring to the leader’s conduct aimed at encouraging their employees to re-examine their beliefs to address old problems in the workplace in an intellectually new manner.

The leader–member exchange (LMX) model describes the leader as the one who provides support, autonomy and responsibility to collaborators in exchange for their commitment [21]. The leader establishes differential relationships of greater trust with some collaborators (i.e., ingroup) as compared to others (i.e., out-group), such that ingroup members are given more responsibilities, freedom in their roles, rewards and attention. Scholars in this field [38,39,40] have identified four main dimensions or types of relational exchanges: contribution, loyalty, affection and professional respect. Specifically, contribution refers to behaviors related to the activities that leaders and collaborators carry on to achieve common goals; loyalty is understood as the mutual support between leaders and subordinates; affection refers to the mutual satisfaction that both parties feel for each other; and professional respect refers to mutual respect for individual professional skills. According to servant leadership [22], the leader shares power and puts the needs of others before their own, helping the collaborators to elaborate emotional experiences and to develop and express their potential. A comprehensive framework proposed by Barbuto and Wheeler [41] identifies eleven dimensions: calling (i.e., the desire to serve and the willingness to sacrifice one’s interests for the sake of others); listening (i.e., the ability to listen, evaluate and accept the ideas and suggestions of others); empathy (i.e., the ability to appreciate the circumstances of others); emotional healing (i.e., the ability to recognize how and when to find a solution (or emotional healing) to disappointed employees) is the most original and unique leadership dimension proposed by this approach as compared to all of the others; awareness (i.e., the ability to observe what is happening by collecting cues from the environment); persuasion (i.e., the ability to influence others without relying on formal authority or legitimate power) is suggested to be more effective than authoritarian leadership; conceptualization (i.e., the leader’s ability to encourage collaborators to use creative processes and promote a more open view (lateral thinking) contributions); foresight (i.e., the ability to predict the future and its consequences, critical to servant leadership); stewardship (i.e., the ability to meet the multilayer contextual needs of both the organization and society as a whole, and provide development opportunities); and community building (i.e., the ability to instill a sense of community and organizational spirit, which produce greater employee commitment and strengthen organizational identity).

Finally, according to authentic leadership, the leader establishes transparent relationships with their own collaborators, promotes a positive climate and self-development, and includes a positive moral perspective characterized by high ethical standards that guide the decision-making process by helping them to face problems with elasticity [20]. The model proposed by Walumbwa, Avolio, Gardner, Wernsing and Peterson [42] draws on and integrates existing theories and research on leadership, emotions, social identity and identification, trust, positive psychology and positive organizational behavior, and includes five different dimensions: self-awareness (i.e., the ability to understand and interpret the surrounding environment and understand the impact it has on other people); relational transparency (i.e., the ability to be authentic and transparent, generating trust in collaborators); internalized regulation (i.e., the ability to self-regulate), balanced processing of information (i.e., the ability of the leader to objectively analyze all relevant elements before making a decision) and positive moral perspective (i.e., the ability to have a positive vision grounded in ethics and integrity).

### 2.2. Group Member Prototypicality and Its Relevance in the Military

The social identity theory of group processes and intergroup relations outlines how people define themselves in terms of the collective attributes of a group they belong to (i.e., social identity or collective self; [7]). Group identification includes a social categorization process, defined as the cognitive grouping of the self as identical to some sets of attributes or group prototypes (i.e., ingroup prototype) in contrast to some other class of stimuli (i.e., out-group). Coherently, group member prototypicality (GMP) could be defined as a process of self-categorization that increases individuals’ identity between the self and ingroup members while simultaneously increasing differences between the self and out-groups [43]. Group prototypical characteristics form an important source of information about social reality, and the more people identify with a group (i.e., define the self in terms of the group identity), the more their beliefs, attitudes and behavior are governed by group membership and, therefore, are uniform and consistent across different members [8]. Consequently, the closer any individual group member comes to the prototypical and normative ingroup position, the more they will be liked by other ingroup members. In turn, this generates the intragroup social attraction and cohesiveness that holds groups together and builds positive interactions among members of an organization or its units. A shared social identity strongly bonds group members to a common prototype, which prescribes the characteristics that gain the greatest group consensus in a given context, and group members’ behavior will be guided by the emergent and distinctive properties of their group rather than by the average of their idiosyncratic opinions [8].

While to our knowledge there are no empirical studies of GMP in the military, the social identity perspective is pervasive in the military as well as the concept of group prototypicality. For example, according to Burroughs and Ruth [44], cohesion in Army units refers to members attractiveness that takes the form of social attraction to fellow group members as group members more than interpersonal attraction because members are liked to the extent that they embody the group itself and are prototypical of unique group features [45]. That is, overall group cohesiveness is impacted more by social attractiveness rather than interpersonal attractiveness. Moreover, as noted above, some leadership dimensions, such as community building of servant leadership theory [41], specifically call into account the social identity perspective in that the leader’s ability to instill a sense of community and an organizational spirit ultimately aims at strengthening organizational identity. Similarly, authentic leadership development builds on social identity and group member prototypicality [20]. Specifically, members who express prototypical group values and aspirations such as integrity, credibility, justice and respect are especially likely to emerge as authentic leaders. This is because prototypical members who model these core values will be viewed as socially attractive and hence influential by other members. Moreover, attributional processes will predispose members to assign leadership qualities to members whose deeds reveal a genuine commitment to core values; yet, authentic leadership emerges if such values as integrity, transparency and justice are widely shared by all members of the collective and there is a positive climate characterized by a commitment to ethical conduct and human development. As such, GMP and social identity principles appear to be intertwined with leadership processes in the military. It is noteworthy that the current military mission’s transformation towards including counterinsurgency and nation-building scopes is exposing soldiers to a) shifting roles with agility (e.g., from combat to assistance), thus challenging their professional social identity and the prototypical features of combat (i.e., warfare competence) or conversely of assistance (i.e., peace-keeping competences), and b) interacting with individuals from cultures that are vastly different from their own on a daily basis, thus challenging their national social identity and the prototypical features of rules and the culture norms of one’s own Army (i.e., in-group) with those of people outside the military (i.e., out-group). Group member prototypicality defined at the level of a work team (or a unit in the military) captures the ”collective” social identities of their members [8] and refers to the extent to which one identifies oneself as a member of a group (e.g., unit) because they perceive themselves as sharing key attributes with other military group members (i.e., in-group) as opposed to civilians or other-army outsiders with features different from the in-group prototype (i.e., out-group). Overall, such social attraction and self-categorization processes (a) crucially engender team cohesion and build team synergy in the military to the extent that members think and act more like the most prototypical members, and (b) contribute to the emergence of leadership and acceptance of authority through identification to the extent that prototypical members are the most informative about the defining characteristics of the group and attract attention, thus becoming influential.

### 2.3. Familiarity at Work: Conceptual Framework and Multistakeholder Approach

Introducing the concept of “familiarity at work” requires addressing two main different, yet interrelated, issues: (a) defining what “familiarity” means in organizational settings, and (b) the existence of different types of organizational stakeholders potentially involved when targeting the subject with whom an organizational member is familiar (e.g., colleagues, leaders, clients). The literature in both areas provides highly heterogeneous and disparate contributions. Below, we briefly review the state of the art in each area and then provide an overarching framework that comprehensively builds upon the existing literature.

When attempting to identify a definition of familiarity in work settings, the literature provides scattered and inconsistent contributions [9] under the general heading of familiarity. For example, *familiarity* could be defined as having a history of interaction [46] or prior experience working with one another [47] and shared task experience (i.e., task familiarity) as opposed to more relation-focused approaches defining familiarity as an interpersonal knowledge of coworkers [48] characterized by deep vs. shallow knowledge about one another [47]. Whether at the dyad or team levels, team literature often defines *team familiarity* broadly as the level of knowledge that team members hold about each other [47,49], the members’ amount of time of joint shared work experience (i.e., a time measure; [50]) and the quality of the team’s relationships and team cohesion between members as well as the quality of the team’s communication [51,52,53]. Finally, Hinds and Cramton ([9]; p. 797) define *situated coworker familiarity* as the “multiplex understanding that coworkers have of their counterparts in relation to themselves and their work together achieved by discussing work but also disclosing personal information, interacting frequently, informally, and deeply into colleagues’ day-to-day lives”. It is noteworthy that some contributions use the term “coworker familiarity” whereas others use the term “team familiarity”.

Additional contributions specifically mention “*work familiarity*” [54], understood as the knowledge employees have of the people they work with in terms of “working practices” (i.e., competences, skills, strengths and weaknesses), whereas [55] defines “*workplace intimacy*” as the feelings of closeness, sharing and knowledge of more affective aspects related to one’s personal life among people at work. It is noteworthy that the literature on workplace intimacy also calls into account the boss–coworker relationship [53,56] beyond the more common coworker–coworker dyad, suggesting that intimate bonds between boss and employee positively influence and facilitate the process of learning, creativity and confidence.

Relatedly, when defining familiarity, the literature also charts the territory by variously proposing what familiarity is not. For example, team familiarity is different from “team tenure”, understood as the amount of time a team member has worked within their team [57], or “perceived proximity” [58], because working nearby might not include interaction, joint experience or shared knowledge. Also, workplace intimacy and familiarity should not be confused with romantic involvement [55] or workplace friendship (which incorporates affective ties; [47]). More importantly, military organizations specifically mention the concept of “familiarity” and its relevance in order to build cohesion and bond among soldiers [6,59]; yet, they also warn against over-familiarity and differentiate familiarity from fraternization, understood as any improper relationship, which can range from business relationships to friendships and romantic relationships [60]. Specifically, while positive personal relationships are marks of solid interactions that tie leader and followers or followers among themselves, perceived unprofessional relationships due to partial or unfair treatment, or resentment behaviors (i.e., fraternization) may destroy discipline, cohesion and/or the chain of command (e.g., [6]). This also applies to other occupational settings, such as healthcare (e.g., [61]) or service businesses (e.g., [62,63]).

Moving to the issue of the subjects involved in interpersonal relations of familiarity in workplaces, the above literature review suggests that research on familiarity is more commonly and primarily focused on dyadic (or group) relationships among coworkers (e.g., [64]), or, rather, among colleagues. Conversely, the literature on familiarity within the boss–employee relationship (e.g., [55]) is still scant; yet, familiarity at work between leader and followers is widely addressed by practitioners through organizational policies (e.g., [6]) and divulgative media (e.g., [63]) in an attempt to draw a line between positive and flawless interactions, and overly inappropriate and unwanted behaviors which downsides for business or morale outweigh the positive of being well-acquainted. While research on familiarity among coworkers is more common and the literature on boss–employee is still limited, studies on familiarity between employees and clients/customers (i.e., external stakeholders) are still absent. Paradoxically, familiarity with clients is massively addressed by organizational policies and divulgative media (e.g., [65]), both in terms of positive and constructive relationships that may help business, as well as potentially improper/adverse workplace behaviors (e.g., fraternization) that may undermine work operations, effective leadership and cohesion of a harmonious work environment (e.g., [62,66]). Hence, the stakeholders involved in the concept of familiarity at work seem to be colleagues and leaders (i.e., organizational members) as well as clients (externals to the organization).

Overall, we can draw the following conclusions. First, more comprehensive definitions of familiarity in work settings seem to include both spending time together as well as knowledge about each other and sharing intimate issues, thus going beyond the mere exposure effect [67]. Second, familiarity at work refers to a state of close relationships but not necessarily of affection like friendship, and should not be confused with romantic involvement, fraternization, mere physical proximity or mere “team tenure” in terms of time spent working together in the past. Third, definitions of familiarity at work in the existing literature usually call into account the relationship between coworkers or among teammates, and to a lesser extent include the boss–employee relationship (e.g., [6,56]), but also the relationship with clients/externals [62,66].

We define *familiarity at work* as a state of close relationships experienced with others at work (e.g., colleagues, leaders, clients/externals), a sense of closeness and self-disclosing intimacy due to mutual sharing of personal matters, and the tendency to actively seek to spend time and interact with others with whom one feels well-acquainted.

### 2.4. Familiarity at Work as a Predictor of Leadership and Social Identity

Scholars and practitioners have debated the consequences of familiarity among individuals at work and whether relational closeness may hurt, rather than benefit, workplace morale and productivity. Consequences of familiarity variously involve the boss–employee interaction as well as the bonds among coworkers and between clients and employees. As we illustrate below, there is theoretical and empirical evidence to suggest competing hypotheses regarding the direction (i.e., positive vs. negative) of familiarity effects on both leadership actions and shared social identity (i.e., GMP).

When considering the potential effects of familiarity with leaders on leadership practices, the literature [55] suggests that traditional leadership theories and management practices tend to distinguish between the public sphere (e.g., the workplace) characterized by control and rationality, and the private sphere (e.g., the home), characterized by spontaneity and intimate personal relationships. However, new models of relational leadership have emerged and more intimate relationships with supervisors (i.e., familiarity with leaders) may foster each other’s understanding, employee well-being and optimal functioning, and ultimately build an effective leadership rooted in relational practice (i.e., to a way of working and managing that is directed toward the welfare of others; [68]). Moreover, practitioners’ contributions (e.g., [69,70,71]) tend to suggest that successful managers must foster camaraderie without becoming too familiar and that, in order for their leadership to be effective, the degree of fraternization with subordinates depends on both personal (e.g., outstanding managers can fraternize with less risk of compromising their leadership) and organizational factors (e.g., type of fraternization or rather, socializing with subordinates at company functions, but always at arms length, can actually foster respect). Familiarity may breed awesome to leadership (i.e., a positive effect), as proposed by a contribution by Spivey [59] in the U.S. Air Force, suggesting that positive familiarity brings trust and efficiency because time is not wasted in the search for knowing each other; yet, neglecting rules and becoming overfamiliar with authorities (i.e., familiarity with supervisors) may jeopardize discipline and bring consequences that far outweigh the benefits (i.e., a negative effect). Similarly, the U.S. Military Code [6] maintains that soldiers of all ranks must feel they belong to the “family” and the Army needs professional, caring interactions because they build vertical bonds that tie leaders and followers, thus fostering and encouraging teamwork and respect for authority.

Based on the above arguments, we predict that:

**Hypothesis** **1a (positive):** *Familiarity at work with leaders will positively predict (1) transformational, (2) LMX, (3) servant and (4) authentic leadership styles*.

However, in the organizational setting, familiarity is often suggested to breed contempt and cause managers to lose control of their authority (i.e., negative effect on leadership) by becoming too familiar with their underlings (e.g., [69,71]). Similarly, good order and discipline are imperative to the success of military organizations and leaders must be counted on to use good judgment, experience and discretion to draw the line between relationships that are “constructive” and those that are “destructive”, thus undermining their authority [6]. While any social relationship among enlisted soldiers of different ranks that reasonably comes within the realm of unit-based social functions and team building is appreciated, the Army prohibits toxic over-familiar relationships for leadership that (1) compromise, or appear to compromise, the integrity of supervisory authority or the chain of command; (2) cause actual or perceived partiality or unfairness; (3) involve, or appear to involve, the improper use of rank or position for personal gain; (4) are, or are perceived to be, exploitative or coercive in nature; and (5) create an actual or clearly predictable adverse impact on discipline, authority, morale, or the ability of the command to accomplish its mission.

Therefore, based on these alternative arguments from the literature, our competing hypothesis predicts:

**Hypothesis** **1b (negative):** *Familiarity at work with leaders will negatively predict (1) transformational, (2) LMX, (3) servant and (4) authentic leadership styles.*

Moving to the potential effects of familiarity with colleagues on leadership practices, the literature [9,70] suggests that interpersonal knowledge among group members about one anothers’ skills, perspectives and interpersonal styles (i.e., familiarity with colleagues) facilitates coordination and the accomplishment of joint tasks (i.e., leadership processes). In the military [6], soldiers of all ranks meet and associate with each other in many settings, both on and off duty, and unit cohesion is crucial for successful joint action and flawless leadership.

However, scholars and practitioners also suggest arguments on the negative effects of familiarity among colleagues on leadership action. Specifically, cohesion is hampered anytime relationships between the unit’s members are unprofessional and compromise the chain of command (i.e., leadership). The appearance of impropriety (e.g., over-familiarity) can be as damaging to morale and discipline as actual misconduct, and how these relationships impact authority, discipline and morale is central to evaluating soldier relationships. Becoming “too close” and overly familiar (e.g., romantic involvement, close friendship) with same-rank soldiers (i.e., coworkers) may distract individuals from work duties and cause involved parties to spend less time developing their relationships with other teammates, which may hurt team unity and undermine leadership effectiveness [60]. Relatedly, there might be the risk that overly familiar relationships among coworkers might end on bad terms, which could impact their ability to effectively work together afterwards, and thus weaken discipline and the leader’s authority. Similarly, organizational policies on relationships among employees (e.g., [63]) suggest that company employees may develop friendships and relationships with other employees—both inside and outside of the workplace—as long as the relationships do not have a negative impact on their work or the work of others, on productivity and the harmonious work environment, and do not interfere with the company culture of teamwork and an employee-oriented management style.

**Hypothesis** **2a (positive):** *Familiarity at work with colleagues will positively predict (1) transformational, (2) LMX, (3) servant and (4) authentic leadership styles*.

**Hypothesis** **2b (negative):** *Familiarity at work with colleagues will negatively predict (1) transformational, (2) LMX, (3) servant and (4) authentic leadership styles*.

When considering the potential effects of familiarity with clients/externals on leadership practices, the literature is still absent. Yet, organizational practices and policies lively address the topic and its regulation. For example, organizational practitioners (e.g., [62]) suggest that some organizations look to build relationships and to know clients really well, and employees having a personal relationship makes sense and is beneficial, just as long as they do not cross the line by becoming overly familiar and share too much information or the relationship turns sour. In turn, this may cause problems for the business and make supervisors (i.e., leadership) uncomfortable with how to manage an employee-client synergy without losing the client. As such, it is suggested to put in place organizational policies that legislate human behavior and the relationships of employees working with clients and discourage relationships of a deeply personal nature with clients in order to prevent the negative impact of over-friendliness on supervisor’s actions and the firm. Similarly, military regulation [6] allows personal relationships between militaries and civilians (i.e., externals) with whom soldiers collaborate and work (i.e., familiarity with clients/externals). However, soldier–civilian intimate relationships and over-familiarity should be avoided and discouraged to the extent that they may raise in the other staff, the impression of preferential or exclusive treatment, which in turn may have a detrimental effect on the morale and efficiency of the group, and related supervisor’s trust and authority (i.e., leadership).

Based on the above arguments in support of familiarity with clients, we predict that:

**Hypothesis** **3a (positive):** *Familiarity at work with clients will positively predict (1) transformational, (2) LMX, (3) servant and (4) authentic leadership styles*.

Alternatively, based on arguments in support of countereffects of familiarity with clients, our competing hypothesis predicts:

**Hypothesis** **3b (negative):** *Familiarity at work with clients will negatively predict (1) transformational, (2) LMX, (3) servant and (4) authentic leadership styles*.

Moving to the role of familiarity at work as a predictor of GMP, the literature [50,72] suggests that high levels of team familiarity enhance team members’ understanding of each other’s expertise, personalities and habits. Familiar group members possess higher interpersonal knowledge and familiarity (i.e., more knowledge about one another’s skills, perspectives and interpersonal styles) and develop a superior meta-knowledge that facilitates interactions and coordination of effort and performs better than groups of strangers [73]. Moreover, teams that work on routine and standardized tasks, as is the case in military settings, may highly benefit from team familiarity, which is a key driver in stimulating the task and social aspects of individual team members [64]. While the role of both familiarity with leaders and familiarity with employees (i.e., organization insiders) in fostering social integration (i.e., the tendency for people who belong to a group to think, feel and act as a group; [74]) may appear straightforward, the role of familiarity with externals (i.e., outsiders) is less immediate. It is noteworthy that the literature [75] suggests that even the relationships group members have with outsiders may increase their visibility (i.e., prototypicality) if they have acquaintances or friends with special resources that the group needs.

While there is currently no study on familiarity at work as a precursor of GMP, based on the above arguments, we may speculate that deep interpersonal knowledge (i.e., familiarity), whether among coworkers and/or between leader and followers and/or between employee and client, tends to bring individuals closer to each other, strengthen an in-group/out-group spirit, thus generating the intragroup social attraction and cohesiveness that holds groups together and builds the shared identity of an organization or an organizational unit (i.e., group member prototypicality; [8]).

Thus, if the above arguments are supported, we would expect the following:

**Hypothesis** **4a (positive):** *Familiarity at work with (a) leaders, (b) colleagues and (c) clients will positively predict GMP*.

The literature on GMP suggests that the closer any individual group member comes to the prototypical and normative ingroup position, the more he or she will *be liked* by other ingroup members and contribute to engendering social attraction that holds groups together. However, to the extent that being overly familiar with someone (e.g., becoming intimate to the point of excluding others, spending too much spare time together) is likely to generate feelings of being saturated with another’s presence or friction in day-to-day interactions, then we might speculate that excessive closeness/familiarity among people at work may cause a sense of *dislike* towards others with whom one interacts and, thus, decrease the perception of social cohesiveness and shared identity among workers (i.e., GMP).

Based on the above arguments, we may pose the following alternative hypothesis:

**Hypothesis** **4b (negative):** *Familiarity at work with a) leaders, b) colleagues and c) clients will negatively predict GMP*.

### 2.5. Does Combat Experience Matter for Familiarity at Work and Its Outcomes?

To contextualize our research, we note that the military literature on leadership, group cohesion, primary group (i.e., peers and leader) and secondary group (i.e., organizational and institutional) relationships (i.e., a proxy of familiarity) and team identification (e.g., GMP), mainly focuses on personnel deployed to *combat* operations, rather than *non-combat* situations. Arguably, this is justified by the still prevailing warfare mission of military organizations that consistently emphasize combat deployment. For example, while in the organizational literature, leadership is often regarded as the glue that binds organizations and catalyzes changes that enable the system to achieve success, the Army regards leadership as an element of combat power and a force multiplier that can enhance warfighting functions [30]. Consistently, most theoretical and empirical contributions focus on leadership in combat environments (e.g., [5,27]). Similarly, the importance of military units’ cohesion has been extensively addressed because of its combat motivation multiplier effects and combat-effectiveness [27]. For example, vertical cohesion (i.e., the positive bond that soldiers have with their leaders) is considered the glue that ensures that the values and norms of lower-level units are consistent with unit interests, and positively affects fighter spirit and combat motivation (ibidem).

While combat deployment is undoubtedly considered the main type of operation in the military, and recently further increased by the war in Ukraine and related geopolitical turmoil, nonetheless, non-combat operations (e.g., humanitarian) are in parallel on the rise. The Modern War Institute at West Point maintains that in this new era of continuous global power competition, the vast majority of the Army’s tasks occur below the threshold of armed conflict [76]. Interestingly, competition is described as a period when “two or more actors in the international system have incompatible interests but neither seeks to escalate to armed conflict” and reflects the changing nature of conflict from the binary war-and-peace model towards a competition continuum of cooperation, competition and armed conflict.

Notwithstanding, few studies focus on non-combat personnel and/or the comparison between combat and non-combat experiences, such as Wilson and colleagues’ study on the impact of war on combat vs. non-combat exposed military personnel lifespan [77]. In general, the literature [5] suggests that studies conducted in combat consistently find that the quality of leader–follower relationships is higher in terms of the degree of cooperation to achieve a common goal, psychological closeness and the extent of caring (i.e., proxies of GMP and familiarity) compared to non-dangerous areas (e.g., non-combat). Based on these findings, we might arguably expect a differential pattern of nomological network among our study variables (i.e., leadership, GMP, familiarity) in the combat vs. non-combat subsamples. Given that we could not find published studies examining the moderating effect of combat experience on the relationship between familiarity at work and GMP as well as leadership, we pose the following research question:

*Research Question:* Does combat experience (combat vs. non-combat) moderate the relationship among familiarity (with leaders, colleagues, externals), GMP and leadership (transformational, LMX, servant and authentic)?

## 3. Method

### 3.1. Participants and Procedure

Surveys were administered to 435 military of the Italian Army from six different units. In the overall sample, 92.4% of respondents identified as male and 7.6% as female. The average age of participants was 31.94 years (*SD* = 8.14), and the average job tenure was 11.59 years (*SD* = 8.49). Thirty-eight percent were enlisted men, while the remaining 62% held a commissioned or non-commissioned officer position. Thirty-one percent were leaders managing a group. While 52.4% indicated at least one experience in operational theatres (i.e., combat group), the remaining 47.6% had no experience in operational theatres (i.e., non-combat group).

Anonymous paper and pencil surveys in Italian were administered during collective sessions held at the military sites involved in the study. An Army Psychological Officer introduced the research team and explained the relevance of the study for the Italian Army. Once consent was obtained, the research team provided information to describe the project, encourage participation and address any concerns from potential participants. The voluntary and confidential nature of participation was secured by collecting surveys back in sealed envelopes.

### 3.2. Measures

Below is a description of measures used to provide data for the current analyses. The transformational leadership, leader–member exchange, servant leadership and authentic leadership scales were translated into Italian from the English version (see Appendix A) using the standard translation–back-translation procedure recommended by Brislin [78]. The correspondence of the original and back-translated items was then verified by the authors.

#### 3.2.1. Familiarity at Work Scale

The development of the Italian version of the Familiarity at Work Scale (the Familiarity at Work Scale is available upon request to the corresponding author) was derived from the above review of the literature and theoretically grounded in a comprehensive definition of familiarity in work settings that include both spending time together as well as knowledge about each other, sharing intimate issues and a state of close relationship that should not be confused with romantic involvement, fraternization or mere physical proximity in terms of time spent working together in the past. Overall, these components altogether contribute to the measurement of familiarity at work, understood as a state of close relationships experienced with others at work (e.g., colleagues, leaders, clients/externals), a sense of closeness and self-disclosing intimacy due to mutual sharing of personal matters and the tendency to actively seek spending time and interacting with others with whom one feels well-acquainted. Consistently, the Familiarity at Work Scale is a three-factor (i.e., familiarity with leaders, familiarity with colleagues and familiarity with externals) measure assessing employee’s familiarity with the three different stakeholders in the workplace. The scale provides the respondents with different work-situated behaviors that indicate their willingness to spend time with others, seek their company and share information on personal matters, and a state of close relationship (not romantic involvement). For each item, participants were asked to indicate how frequently they experienced the given situation with leaders, colleagues and externals. A sample item is: “When I take a break at work, I often choose to spend it with …”. Respondents were asked to indicate how often the work situation presented in the item occurred, respectively, with their leaders, colleagues, and externals. The response format was a 5-point Likert scale ranging from 1 (Never) to 5 (Always). Given the scale format, the six items can be classified as common stem items. The scale format allowed us to compute three different independent scores, respectively, for familiarity with leaders, familiarity with colleagues and familiarity with externals.

#### 3.2.2. Group Member Prototypicality

Group member prototypicality was assessed using five items of the Italian version of the Team Identification scale from Cicero, Pierro, and van Knippenberg [79]. A sample item is the following: “When I talk about my work team, I usually say “we” rather than “they”. Responses were rated on a 6-point scale ranging from 1 (strongly disagree) to 6 (strongly agree).

#### 3.2.3. Transformational Leadership

Transformational leadership was measured using fifteen items from the Transformational Leader Behaviors Scale [80] assessing a leader’s charisma, leading by providing a model and individualized support and fostering group goal acceptance. Responses were made on a 5-point frequency scale from 1 (Never) to 5 (Always). A sample item is the following: “Provides a good model for me to follow”.

#### 3.2.4. Leader–Member Exchange

Perceptions of leader–member exchange between respondents and their leaders were measured using seven items from the leader–member exchange scale [21] assessing leader’s respect, trust and obligation. Responses were made on a 5-point frequency scale from 1 (Never) to 5 (Always). A sample item is the following: “My leader understands my job problems and needs well”.

#### 3.2.5. Servant Leadership

Servant leadership was measured using thirteen items from the altruistic calling, emotional healing and wisdom dimensions of the Servant Leadership Scale [41]. Responses were made on a 5-point frequency scale from 1 (Never) to 5 (Always). A sample item is: “My leader is good at helping me with my emotional issues”.

#### 3.2.6. Authentic Leadership

Authentic leadership was measured using eight items from the Authentic Leadership Questionnaire [42] assessing a leader’s self-awareness, relational transparency, internalized regulation (i.e., authentic behavior) and balanced processing of information. Responses were made on a 5-point frequency scale from 1 (Never) to 5 (Always). A sample item is the following: “My leader seeks feedback to improve interactions with others”.

#### 3.2.7. Control Variables

Given our study included leadership dimensions, we controlled for the effects of managerial position (i.e., hierarchy level) on leadership styles [81].

### 3.3. Statistical Procedures

In order to maximize the reliability and parsimony of our structural equation model, item parcels were created for construct measures with more than three items (i.e., familiarity at work, GMP, authentic leadership, transformational leadership, LMX and servant leadership). We followed Little, Cunningham, Shahar, and Widaman’s [82] recommendations and sequentially assigned items based on the highest to lowest item-to-construct loadings/correlations to create three item parcels per construct. Subsequent analyses were conducted with M*plus* 8.7 [83] using the parceled items and maximum likelihood estimation with robust standard errors.

Next, we performed separate confirmatory factor analyses (CFA) for the combat and non-combat samples on the nine continuous constructs. Second, we used the multiple-group CFA to determine if ratings of items demonstrated invariance across the combat and non-combat samples, respectively, using the procedure proposed by Cheung and Rensvold [84]. In short, this procedure involves comparing progressively more constrained models that test for measurement invariance: configural invariance (equality for form), metric invariance (equality for loadings) and scalar (equality for intercepts). When there is some support for measurement invariance [85], structural invariance (equivalency for factor variances, factor covariances and factor latent means) may be examined. Once measurement invariance and structural invariance are supported, the moderation effect of one’s combat experience (i.e., combat vs. non-combat) may be explored.

## 4. Results

### 4.1. Descriptive Statistics and Correlations

Table 1 presents the descriptive statistics, scale reliabilities and intercorrelations among the study variables, both for the combat and non-combat subsamples.

### 4.2. Test of Measurement Model on the Total Sample

In order to test our measurement model, we performed an initial confirmatory factor analysis consisting of the hypothesized eight latent variables (i.e., familiarity with leaders, familiarity with colleagues, familiarity with externals, GMP, authentic leadership, transformational leadership, LMX, servant leadership) and their respective item-level indicators. We then compared the fit of the hypothesized model (A0) with the first plausible alternative model that combined the three forms of familiarity into a single factor (A1) and a second plausible alternative model that combined authentic and transformational leadership into a single factor (A2). The fit indices for the base model (A0) were as follows: χ^2^ (224, N = 435) = 584.725, *p* < 0.001, RMSEA = 0.061 (0.055; 0.067), CFI = 0.950, SRMR = 0.051. The fit indices for the first alternative model (A1) were as follows: χ^2^ (237, N = 435) = 1652.046, *p* < 0.001, RMSEA = 0.117 (0.112; 0.123), CFI = 0.804, SRMR = 0.100. The Chi-square difference tests between the base model and the first alternative model showed a significant (*p* < 0.001) Δχ^2^ (13, N = 435) = 1067,321. The fit indices for the second alternative model (A2) were: χ^2^ (231, N = 435) = 636.629, *p* < 0.001, RMSEA = 0.064 (.058; 0.069), CFI = 0.944, SRMR = 0.052. The Chi-square difference tests between the base model and the second alternative model showed a significant (*p* < 0.001) Δχ^2^ (7, N = 435) = 51,904. Both alternative models (A1, A2) showed worse fit indices in comparison to the base model (A0). Based on model fit indices comparison and the Chi-square difference tests of the nested models, the best fitting model appeared to be the base model. Finally, following Fornell and Larcker’s [86] recommendation for the assessment of discriminant validity, we calculated the average variance extracted (AVE) of the three sub-dimensions of the familiarity at work scale and compared the square root of each familiarity sub-dimensions AVE with their correlations among each other as well as with all the other latent constructs of our model (i.e., GMP, authentic, transformational, LMX, servant). If the square root of each construct’s AVE shows a higher value than the correlations with the other latent constructs, discriminant validity is supported. Our results show that the squared AVE respectively for familiarity with leaders (0.85), familiarity with colleagues (0.83) and familiarity with externals (0.88) display higher values than the correlations of each of the three dimensions among each other, as well as compared to the correlations (reported in the CFA) of each of the three dimensions with all the other latent constructs. (For the sake of parsimony, we do not report the full output. Results are available upon request to the corresponding author.) Taken together, these results provide support for the internal and discriminant validity of the three-factor familiarity at work scale and the distinctiveness among all of the eight study variables. Therefore, the following analyses were performed on the hypothesized eight-factor model.

### 4.3. Goodness of Fit for the Single Groups

Prior to conducting multiple-group analyses to test our hypotheses, we examined the goodness-of-fit values of the CFA models separately for the combat and non-combat samples. As shown in Table 2, the values for both the combat and non-combat samples showed an excellent fit to the data.

### 4.4. Test of Measurement Invariance across Groups

Table 2 shows the results of the analyses for invariance testing. Each of the three invariance levels (i.e., configural, metric, scalar) provided good fit for models M1, M2 and M3, and the decrease in the CFI value was less than 0.01 for configural (M1) and metric (M2) invariance comparison. When constraints on thresholds were introduced to test for scalar invariance (M3), the model still showed a good fit but did not satisfy the full scalar condition. Thus, the latent variables of our study shared the same meaning (i.e., equality of factor variances and co-variances) across the combat and non-combat samples, and structural effects may be appropriately compared across groups.

### 4.5. Multigroup Structural Equation Models

In order to test our hypotheses and research question on the moderation effect of one’s combat experience (i.e., combat vs. non-combat), we followed three steps [87]. First, we examined separately the relative fit of a structural regression model for combat and non-combat samples, followed by a single analysis across both groups without any constraints. Then, we tested the structural model for invariance across groups by adding constraints to all coefficients.

The results of multigroup structural equation model analyses are shown in Table 2 and are as follows. Values for both the combat military sub-sample (M4) and non-combat (M5) military sub-sample showed good fit to the data. Additionally, fit indices of the structural equation model across the two groups (i.e., combat, non-combat) without constraints (M6) showed good fit. Once invariance constraints across groups were imposed (M7), fit indices did not worsen and there was not a significant decrement in model fit (Δχ^2^(20, N = 435) = 20.964, *p* < 0.001). Overall, this indicated an invariant pattern of relationships among variables for combat and non-combat subsamples. In other words, these findings answered our research question by suggesting that combat experience does not moderate the relationship between familiarity, GMP and leadership styles. As such, all structural paths were interpreted by running a final model on the total sample of military personnel. The final model (M8) showed an excellent fit to the data: χ^2^ (234, N = 435) 528.693, *p* < *0*.001, RMSEA = 0.054 (0.048; 0.060), CFI = 0.960, SRMR = 0.055.

**Figure 2:**
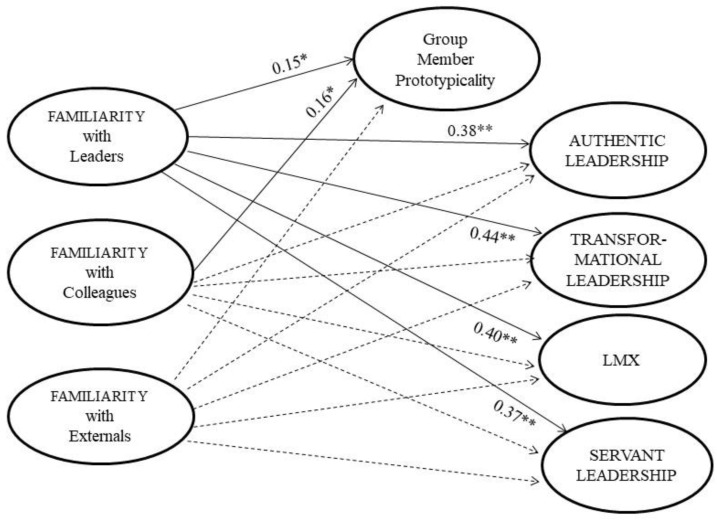
Standardized structural coefficients for the final structural model. Note. ** *p* < 0.01, * *p* < 0.05; dotted lines are statistically non-significant estimates.

As can be seen in Figure 2, familiarity with leaders predicted GMP (0.15, *p* < 0.05), authentic (0.38, *p* < 0.01), transformational (0.44, *p* < 0.01), LMX (0.40, *p* < 0.01) and servant (0.37, *p* < 0.01) leadership. Hence, Hypotheses 1a and 4a were fully supported. Familiarity with colleagues predicted GMP (0.16, *p* < 0.05) and did not predict any of the leadership styles. As such, Hypothesis 4a was supported but not Hypothesis 2a. Familiarity with externals did not exert significant effects on GMP nor on any of the leadership styles. Thus, Hypotheses 3a and 3b were not supported. Finally, the control variable (managerial position) did not exert significant effects on GMP or on any of the leadership styles. Overall, the model explained 7% of the variance in GMP, 14% of authentic, 17% of transformational, 15% of LMX and 12% of servant leadership styles.

### 4.6. Post Hoc Analyses for Quadratic Effects

**Figure 3:**
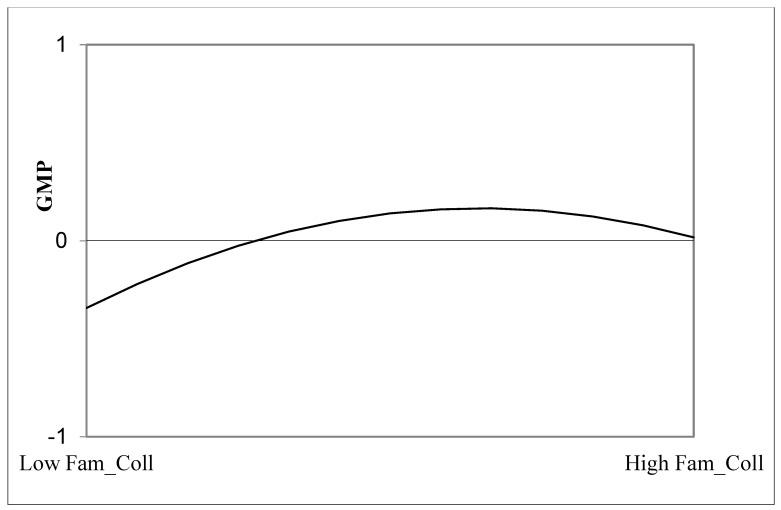
Quadratic effect of familiarity with colleagues on GMP. Note: Fam_coll = familiarity with colleagues; GMP = group member prototypicality.

Post hoc analyses focused only on the significant effects of the structural model. In order to assess the linear vs. quadratic nature of the *significant* effects of familiarity on GMP and leadership styles, we ran a set of polynomial quadratic regression analyses, assessing (a) the quadratic term of familiarity with leaders as a predictor, respectively, of GMP, authentic, transformational, LMX and servant leadership as dependent variables, and (b) the quadratic term of familiarity with colleagues as a predictor of GMP. Results showed a non-significant quadratic effect of familiarity with leaders on GMP, authentic, transformational, LMX and servant leaderships, thus demonstrating the linear nature of the tested effects. Conversely, results showed a significant quadratic effect (*β* = −0.988, *F*(2, 430) = 9.892, *p* < 0.01) of familiarity with colleagues on GMP. In order to evaluate the form of the quadratic effect, we used the Excel plotting program developed by Dawson [88]. As can be seen in Figure 3, the relationship between familiarity with colleagues and GMP demonstrated an inverted-U shape. Specifically, militaries with very low levels of familiarity with colleagues or extremely high levels of familiarity with colleagues reported lower levels of GMP compared to militaries with moderate levels of familiarity with colleagues who reported higher levels of GMP. Interestingly, the right-hand section of the plot shows that at very high levels of familiarity with colleagues, GMP begins to decrease (i.e., a slightly negative relationship).

## 5. Discussion

The mission of military institutions worldwide has increasingly extended beyond conventional warfare and the binary war-and-peace model courses of action [10,76]. Leaders in the military are now often required to shift roles and render humanitarian assistance, and be involved in infrastructure project management [11]. Consistently, they need to demonstrate proficiency in both conventional warfare as well as relations and emotion management. For example, in Italy, a general of the Army was asked to render support to civilians during the COVID-19 pandemic and was nominated as Commissioner for the vaccination program of the Italian population [89]. Similarly, despite the globally arising military mobilization due to the war in the Ukraine, soldiers and their leaders are also facing the challenges of providing humanitarian support to refugees and civil populations.

The aims of the present study were to (a) propose a multifaceted conceptualization, and accompanying scale, of familiarity at work with different stakeholders that targeted not only an employee comfort zone in their relationship with leaders and colleagues (i.e., organizational members) but also with roles external to the organization; (b) examine, in a military context, familiarity at work with leaders, colleagues and externals (e.g., civilians, military from allied nations) as a relational antecedent of the social identity of organizational members (i.e., GMP) and leadership styles of their leaders grounded in relational and emotional factors (i.e., transformational, authentic, leader–member exchange, servant); and (c) examine the moderating role of combat vs. non-combat experience of military personnel on the nomological network of the study variables.

Our findings provide preliminary support for the internal and discriminant validity and reliability of the newly developed multidimensional familiarity at the work scale. Moreover, results on the multigroup SEM analysis showed a non-significant effect of the moderating role of combat experience on the hypothesized nomological network, thus indicating that the pattern of association among familiarity at work, GMP and leadership works the same for both soldiers with and without combat experience. Specifically, our findings on the overall sample suggest that *familiarity with leaders* positively predicted GMP and all leadership styles as well. That is, the more military personnel (either combat or non-combat) feel close to their leader, share with them their personal matters, and intentionally spend spare time or breaks with them (i.e., familiarity with the leader), the more they report that they identify with their working group, feel the team success as their own, use language artifacts such as “we”, rather than “they” when speaking of their team, and feel personally insulted when someone criticizes the group (i.e., GMP). Additionally, the higher the familiarity with the leader, the higher the perception of (a) a leader encouraging teamwork, leading by example and respecting others’ personal feelings (i.e., transformational leadership); (b) a leader adequately acknowledging a soldier’s potential and an efficacious relationship with one’s leader (i.e., LMX); (c) a leader being a serious person, behaving coherently with their opinions and principles, and seeking constructive feedback (i.e., authentic); and (d) a leader postponing themselves for what is best for the soldier, standing as a landmark in case of traumatic experiences and providing support in case of emotional issues (i.e., servant). Interestingly, while the familiarity effect was higher for transformational leadership, followed by LMX, authentic and servant, all effects were similarly high suggesting that familiarity transversally increases all four leadership styles that diversely include relational and emotional factors. Indeed, we specifically focused on leadership models that explicitly embed the relational factor in their conceptualization. Hence, all leadership models included in our research are linked to the relational experience of familiarity in organizations. However, the higher effect for transformational leadership seems to comport with the primacy of this leadership style in the military literature (e.g., [5]). This is likely due to its focus on enthusiastically involving followers towards a common goal and motivating them to achieve it, which mirrors the pragmatical priority to succeed (i.e., win) in the military.

As for *familiarity with colleagues*, our findings suggest that the more combat or non-combat military personnel feel close to their coworkers and intentionally spend spare time or breaks with them, the more they report that they identify with their team and its success (i.e., GMP). Yet, our results also demonstrate a curvilinear relationship among these variables suggesting that at very high levels of familiarity with colleagues, the soldiers’ identification with their team (GMP) starts to decrease. As such, the optimal level of soldiers’ identification with their team is associated with moderate levels of familiarity among coworkers. Moreover, familiarity with colleagues is not associated with any of the leadership variables, thus suggesting that a close relationship among coworkers is independent of the soldiers’ perception of their leader’s competencies and personal characteristics. This finding likely suggests the primacy of vertical bonding (e.g., leader–follower familiarity) rather than horizontal bonding (e.g., familiarity among peers) for leadership’s action [90]. Finally, our results suggest that feeling close to externals and intentionally spending spare time or breaks with them (i.e., *familiarity with externals*) does not predict soldiers’ identification with their team (GMP) nor their perception of their leader’s actions and competencies (leadership factors). Overall, two key findings emerge from our study. Familiarity with leaders has a positive linear effect (i.e., more is better) on both GMP and leadership, thus providing support for the relevance of vertical cohesion for both team identification (i.e., GMP) and leadership processes. Second, while familiarity with a leader has a linear and positive effect on both social identity and leadership outcomes, familiarity with colleagues predicts only team identification and exerts a curvilinear effect, such that the positive relationship with GMP turns negative at very high levels of familiarity (i.e., too-much-of-a-good-thing effect; [91]).

### 5.1. Theoretical and Practical Implications

Our findings have implications for the existing literature in the areas of familiarity at work, social identity, GMP and leadership. First, we extend the literature on familiarity at work by providing clear theoretical and conceptual distinctions between familiarity and other similar (e.g., team tenure), as well as different (e.g., fraternization) concepts, thus contributing to clarifying an area of the literature that has been fraught with conceptual overlap and ambiguity. Moreover, we expand the previous literature by focusing not only on the most widespread concept of familiarity among coworkers (e.g., team familiarity), but also reviewing organizational practices and practitioners’ contributions aiming at regulating employees’ closeness with their supervisors (i.e., familiarity with leaders) and external stakeholders, such as business clients or civilians (i.e., familiarity with externals). Finally, our study answers the call (e.g., [64]) for further investigation of potential counterintuitive outcomes (i.e., curvilinear effects) of familiarity. Given our stratified and multistakeholder approach to familiarity, our study unfolded curvilinear effects of familiarity among coworkers on GMP; yet, it showed linear effects of familiarity with leaders on leadership styles. While the overall findings are encouraging, it should be noted that they only represent a first step, providing initial evidence of the theoretical and practical relevance of considering multiple foci of familiarity and examining the linear/curvilinear effects of each foci on the group as well as leadership outcomes of familiarity. It is noteworthy that the results of the measurement model CFA provided clear empirical support for our proposed three-dimensional conceptualization of familiarity at work, and related operationalization. Hence, our proposed multidimensional conceptual framework, and accompanying scale of familiarity at work rooted in a stratified and multiple stakeholder perspective of organizational social context (i.e., leaders, colleagues and externals), may serve as a complementary contribution to ongoing research and the literature on workplace closeness, which has tended to alternatively focus on either horizontal peer bonding (e.g., colleagues) or vertical bonding (i.e., leader).

Second, we extend research on leadership, and military leadership in particular, by testing familiarity at work as a predictor of leadership styles. As noted above, relational bonding at work could be considered on a continuum, ranging from mere physical exposure to, or interpersonal knowledge of, others (e.g., [67]) up to the opposite pole of deep intimacy and affective involvement (e.g., [47]), with familiarity capturing a balance between the two and standing as an indicator of an intermediate relational depth. While the literature has extensively addressed the role of relational factors on leadership (e.g., cohesion; [27]), the current study is the first to specifically focus on familiarity. On the one hand, we reconciled the conflicting approaches to workplace bonding/familiarity by testing competing hypotheses (positive vs. negative) on the familiarity–leadership link. Our findings on the positive linear relationship between familiarity with leaders and leadership styles (i.e., transformational, leader–member exchange, servant, authentic) provide an initial answer to the widespread debate on whether familiarity at work may breed awe or contempt (e.g., [59,69]) and suggest a more-is-better effect. Moreover, our findings combined with a focus on leadership styles rooted in a leader’s management of relational and emotional processes answer the call for research on the relational and emotional skills of leaders’ actions in the military [5,11]. On the other hand, our results on the role of familiarity with leaders stand out from the other non-significant sources of familiarity (i.e., colleagues, externals) and comport with similar findings in the literature suggesting that in the military, horizontal cohesion is not unimportant; yet, leadership and vertical cohesion are simply the key elements in combat motivation and effectiveness [27]. As such, we add to the leadership research by simultaneously testing the role of multiple stakeholders’ foci of familiarity at work, thus bringing informational power to scholars and practitioners on complex and stratified relational dynamics affecting leadership processes in organizations.

Finally, our study contributes to social identity and the GMP literature by testing familiarity at work as a predictor of team member identity (i.e., GMP). Our finding of familiarity with leaders positively predicting soldiers’ identification with their unit’s prototypical features (i.e., GMP) comports with the previous literature [90,92] suggesting that vertical cohesion (i.e., leader–followers bonding) fosters soldier’s tendency to identify themselves as a member of the group and sharing key attributes with teammates. Moreover, our study further extends the literature by demonstrating that familiarity with colleagues also predicts soldiers’ GMP. However, while familiarity with leaders demonstrated a positive linear relationship with GMP, familiarity with colleagues was curvilinear, such that this relationship is initially positive but turns negative as familiarity increases further (i.e., too-much-of-a-good-thing effect). Our findings contribute to the contemporary call for further investigation of the potential counterintuitive effects of familiarity on team relationships [64]. It is noteworthy that the inflection point where GMP starts turning slightly negative is located at very high levels of familiarity with colleagues, and the right side of the bell-shaped curve starts bending very gradually in comparison to the very steep rising trend of the left side of the curve (i.e., the positive relationship between GMP and familiarity with colleagues). That is, the counterproductive effect of over-familiarity among soldiers on their identification with their unit shows only at very high levels of intimate bonding. This lends support to the view that familiarity among coworkers may bring awe in workplaces (e.g., [59]), but should be disciplined in order to avoid the downsides of excessive closeness, such as the “near or far” type of conflict [93] or miscommunication and distractions [94], weaken team cohesion [6,63]. Moreover, our finding comports with the previous management literature suggesting that seemingly puzzling results in key areas of organizational behavior (e.g., leadership) may be explained by the meta-theoretical perspective provided by the too-much-of-a-good-thing effect [95].

From a practical standpoint, our findings have several implications. First, our results on the positive and linear effects of familiarity with leaders (i.e., bonding with one’s leaders) on both leadership styles and team identification (GMP) reveal the more-is-better situation, such that nurturing vertical cohesion is beneficial for both team identification and leader’s action. Interestingly, the role of the leader and the sense of closeness that they engender in their followers (i.e., a relational factor) is key for strengthening followers’ (a) sharing of a common identity that facilitates shared goals achievement, and (b) appreciation of leader’s attention to relational and emotional aspects of leadership action (i.e., facilitation processes) that inextricably co-exist along with task fulfillment requirements (i.e., production processes). Whether in the realm of civilian management focused on leadership as the glue that binds organizations and enables the business system to achieve success or military management positing leadership as an element of combat power that may enhance warfighting functions [30], leaders are the key to influencing other people in order to drive and facilitate conjoint working activities [26]. According to Augier, Knudsen and McNab [96], the field of organization studies may learn from a closer study of behaviors in military organizations, and the cross-fertilization between the two fields of study is needed for advancing both scholarship and practical aspects of organizing in military and non-military organizations. As such, our pilot study grounded in military and non-military contributions and tested in a military context may represent an initial attempt at mutually advantageous spillover of knowledge between the two fields. Organizations are advised to increase training for leaders on vertical cohesion and develop their awareness of how they manage their intimate bonding with followers and how such a bond may impact the followers’ in-group/out-group dynamics and their perception of the leader’s success in juggling tasks, as well as relational and emotional requirements. In the military, consistent with the 2018 Joint Concept for Integrated Campaigning [76] that replaced the binary war-and-peace model, intervention programs might fruitfully provide leaders with tools to help them augment self-awareness of emotional processes and relational dynamics. For example, the practical group space model used in practice-based trainings describes three fundamental conflict dimensions along which groups typically develop, such as hierarchy (i.e., power), affiliation and intimacy [97]. While training on hierarchy conflict allows gaining knowledge and self-awareness on tensions arising from “top or bottom” issues in a social context, exploring affiliation-related conflict may help leaders to become mindful of friction due to “in-group or out-group” feelings and relational dynamics (e.g., GMP). Finally, intimacy conflicts allow for the awareness of friction caused by “near or far” issues experienced in social relations and the regulation of closeness and distance among group members (i.e., familiarity). Altogether, they provide the training for leaders with an overarching framework capturing the complexities of dealing with vertical bonding in organizational settings, ranging from the interface between vertically different roles (hierarchy) to belongingness and social self-categorization problems (affiliation), up to the difficulties of reaching a balance in relational closeness/distance of interactions at work.

Second, our findings on the curvilinear bell-shaped relationship between familiarity with colleagues (i.e., horizontal bonding) and GMP bring to the forefront the relevance of regulating social exchanges among employees at work, yet with the intrinsic difficulty of disciplining “spontaneous” demonstrations of social attraction and preferences that pragmatically contribute to task fulfillment. On the one side, organizations are advised to facilitate the social and “family” cohesion environment by developing policies that help incumbents actively manage the interplay between public and private life domains and the potential downsides of blurring the boundaries. Towards this end, the right balance between close and too close among coworkers is a matter for any company to set the boundaries between roles and the perimeter of interpersonal “do’s and don’ts”, and doing it consistently with the companies’ specific mission and business as well as corporate identity and culture [6,63]. On the other side, drawing upon military leadership suggests that in order to ensure that the right things happen at the right time in working units, leaders are also responsible for establishing the policies and procedures necessary to support the development of cohesive teams [98]. For example, if a subgroup indulges in intimate bonding to the point of setting itself apart, leaders cannot afford to have their ability to accomplish the mission, as a group, negatively influenced by a subgroup and are responsible for taking action and explaining the impact that exclusive horizontal bonding is having on unit cohesion. As such, organizations are warned on the relevance of leaders’ training on team building and management techniques that facilitate the development of informal social group norms in line with organizational desires and the accomplishment of goals or missions set by the organization.

### 5.2. Limitations and Future Directions

While our study is an important first step in demonstrating the role of familiarity at work as a key relational factor in prompting both employees’ identification with their team and leadership styles attentive to relational and emotional factors, it also suffers from some limitations that should be addressed in future research efforts. First, we tested our hypotheses using cross-sectional data, which may raise a concern about the likelihood of common method variance bias [37] as an explanation for our observed relationships. Future studies may contribute to minimizing this bias and demonstrate the causal relationship of the posited nomological network using cross-lagged data that introduce a temporal distance between predictors and outcomes. Longitudinal data where the predictors are measured at Time 1 and outcomes are measured at Time 2 would strengthen these findings. Moreover, additional outcomes (e.g., team effectiveness, team well-being) measured at Time 3 may further strengthen our conclusions by allowing for the examination of the interplay between within-person processes (i.e., familiarity) and leadership dynamics using latent growth curve models.

Second, we tested our hypotheses solely using self-reported data. While secondary source data would be of limited value when assessing constructs such as perceived familiarity and employee identification with their team for which the individual is arguably the best informant, future research could obtain additional multisource data of leadership styles through supervisor’s inquiry (i.e., self- vs. hetero-assessment). Moreover, the Time 3 assessment of team outcomes might include objective indicators of team emotional and mental well-being (e.g., archive records).

Third, while our data was drawn from a large military sample representing a reasonable variety of units and combat (as well as non-combat) experience, it was nonetheless a convenience sample from one national context. Hence, our findings might arguably be affected by self-selection bias. Future research on larger samples including different military forces and cross-country studies based on data from different nations might corroborate our findings and increase the likelihood of generalizability of the research findings and applications. Additionally, this would comport with the literature on the relevance of cultural intelligence for leadership, whether considered as a force multiplier in the military or a cross-cultural facilitator between business boundaries with profit as the major incentive in the civilian management area [99].

Fourth, the current study is the first to demonstrate the effect of familiarity at work with multiple stakeholders in prompting soldiers’ identification with their team and leadership processes including management of emotional and relational processes; yet, this does not apply to familiarity with externals. Moreover, leadership in the military is made unique by the dangerous situations that shape the military (e.g., there are no substitutes for leadership, and followers are indoctrinated into the chain of command and control; [5]). As such, future studies could target occupational settings other than the military in order to assess the generalizability of our findings as well as target organizations operating in the service sector wherein the role of clients is crucial for their mission. Moreover, these partial predictive effects indicate a need to include other potential explanatory/mediating variables. Consistent with Laurence’s [11] recommendation to consider relational and emotional factors in military leadership studies, the current framework could be further expanded by examining the mediating role of emotional contagion, defined as the tendency to automatically absorb emotions from others with whom one interacts at work and that could be assessed in association to multiple organizational stakeholders such as leaders, colleagues and externals [100]. Hence, it represents a combination of emotional dynamics intertwined with different social pathway interactions at work. Inclusion of emotional contagion in future research would greatly aid our understanding of how intense and close interactions at work (i.e., familiarity) may translate into effective emotions and relation management on the part of the leader, particularly in light of the complex co-existence of different horizontal (i.e., colleagues, clients) and vertical (i.e., leaders) sources of social exchanges at work.

Finally, while our results suggesting a positive linear relationship between familiarity with leaders and all leadership styles, as well as a curvilinear relationship between familiarity with colleagues and GMP, are promising, an additional venue for advancing the literature on familiarity at work and its outcomes points at incorporating the study of how contextual effects of organizational processes, such as organizational culture, norms and values (e.g., [5,44,101]), influence and shape the relational environment of employees as the bedrock of their experience of leadership processes and unit identification. Toward that end, future studies considering possible organizational differences and taking a multilevel modeling approach should target employees nested within a large number and wide variety of organizations [102].

## Figures and Tables

**Table 1:** Descriptive statistics, correlations and reliabilities.

Variable	M	*SD*	1	2	3	4	5	6	7	8	9
Familiarity with Leaders	1.91 (1.96)	0.75 (0.70)	0.87 (0.81)	0.25 **	0.38 **	0.19 **	0.40 **	0.42 **	0.41 **	0.39 **	0.13 *
2. Familiarity with Colleagues	3.56 (3.31)	0.91 (0.92)	0.18 *	0.88 (0.87)	0.03	0.20 **	0.08	0.05	0.04	0.002	−0.23 **
3. Familiarity with Externals	1.64 (1.81)	0.85 (0.90)	0.21 **	0.11	0.91 (90)	0.10	0.15 *	0.10	0.14 *	0.14 *	0.06
4. GMP	4.85 (5.06)	0.94 (0.95)	0.22 **	0.13	0.01	0.75 (0.75)	0.22 **	0.26 **	0.21 **	0.19 **	0.000
5. Authentic Leadership	3.07 (3.05)	0.86 (0.87)	0.447 **	0.07	−0.01	0.25 **	0.88 (0.90)	0.87 **	0.81 **	0.80 **	0.02
6. Transformational Leadership	3.33 (3.29)	0.91 (0.91)	0.46 **	0.09	−0.01	0.32 **	0.84 **	0.93 (0.93)	0.83 **	0.85 **	0.03
7. LMX	3.12 (3.12)	1.02 (1.0)	0.44 **	0.10	0.01	0.30 **	0.79 **	0.85 **	0.91 (0.92)	0.81 **	0.05
8. Servant Leadership	2.95 (2.98)	0.92 (0.90)	0.44 **	0.04	0.01	0.25 **	0.77 **	0.84 **	0.81 **	0.93 (0.93)	0.03
9. Position (leader)	1.09 (1.50)	0.299 (0.50)	0.003	−0.21 **	−0.01	0.01 *	0.13	0.13	0.14 *	0.09	-

Note: * *p* < 0.05; ** *p* < 0.01. Cronbach’s alpha reliability coefficients are on the diagonal. Descriptive statistics and reliabilities for the combat subsample are in parentheses. Correlations below the diagonal are for the non-combat subsample and correlations above the diagonal are for the combat subsample. LMX = leader–member exchange.

**Table 2:** Results of tests for invariance across combat and non-combat subsamples.

	Model Fit					Model Difference			
Models(M)	χ^2^	*df*	RMSEA	CFI	SRMR	ΔM	ΔCFI	Δ*df*	Δχ^2^
Model*_Combat_*	493.092	224	0.073	0.932	0.065				
Model*_Non-combat_*	384.464	224	0.059	0.956	0.055				
M1: Configural	877.605	448	0.066	0.943	0.060				
M2: Metric	896.813	464	0.065	0.943	0.063	M2-M1	0.000	16	19.208
M3: Scalar	928.276	480	0.066	0.941	0.063	M3-M2	−0.002	16	31.463
M4: SEM for *Combat*	457.098	234	0.065	0.945	0.067				
M5: SEM for *Non*-*combat*	352.863	234	0.050	0.968	0.057				
M6: Single analysis across groups with no constraints	932.308	494	0.064	0.943	0.078				
M7: Single analysis across groups with constraints	953.272	514	0.063	0.943	0.080	M7-M6	0.000	20	20.964
M8: Single analysis Total sample	528.693	234	0.054	0.960	0.055				

Note: At each step in the sequence of invariance tests, all earlier constraints remain in place. RMSEA = root-mean-square error of approximation; CFI = comparative fit index; SRMR = standardized root-mean-square residual.

## Data Availability

The data of the present study are unavailable as participants did not provide their permission to share.

## Appendix A

**Table A1:** Translated Italian Version of the English Leadership Scales Used in the Research.

Italian Version	English Version
Transformational Leadership	
Agisce senza considerare i miei sentimenti	Acts without considering my feelings. (R)
2. Disegna un quadro interessante del futuro per il nostro gruppo	2. Paints an interesting picture of the future for our group.
3. Gestisce “facendo”, piuttosto che semplicemente “parlando”	3. Leads by “doing,” rather than simply by “telling”.
4. Rappresenta per me un buon modello da seguire	4. Provides a good model for me to follow.
5. Si comporta in modo sollecito verso i miei bisogni personali	5. Behaves in a manner thoughtful of my personal needs.
6. Comprende chiaramente dove stiamo andando	6. Has a clear understanding of where we are going.
7. Favorisce la collaborazione tra gruppi di lavoro	7. Fosters collaboration among work groups.
8. Ispira gli altri con i suoi piani per il futuro	8. Inspires others with his/her plans for the future.
9. Riesce ad ottenere che gli altri si impegnino nel suo sogno	9. Is able to get others committed to his/her dream.
10. Incoraggia i collaboratori a fare “gioco di squadra”	10. Encourages employees to be “team players”.
11. E’ sempre in cerca di nuove opportunità per l’organizzazione	11. Is always seeking new opportunities for the organization
12. Ottiene che il gruppo lavori su un obiettivo comune	12. Gets the group to work together for the same goal.
13. Gestisce dando l’esempio	13. Leads by example.
14. Sviluppa un atteggiamento ed uno spirito di gruppo tra i collaboratori	14. Develops a team attitude and spirit among employees.
Leader–Member Exchange	
Di solito so quanto il mio capo è soddisfatto/a di cosa faccio	I usually know how satisfied my leader is with what I do
2. Il mio capo comprende bene i miei problemi e bisogni lavorativi	2. My leader understands my job problems and needs
3. Il mio capo riconosce adeguatamente il potenziale	3. My leader recognizes my potential
4. Indipendentemente dall’autorità formale che il mio capo ha raggiunto nella sua posizione, userebbe il suo potere per aiutarmi a risolvere i miei problemi	4. Regardless of how much formal authority my leader has built into his/her position, s/he would use his/her power to help me solve problems
5. Ancora, indipendentemente dall’autorità formale che il mio capo ha, mi aiuterebbe a superare una difficoltà a sue spese	5. Again, regardless of the amount of formal authority my leader has, he/she would “bail me out” at his/her expense
6. Ho abbastanza fiducia nel mio capo al punto da prendere le sue difese e giustificare una sua decisione nel caso in cui lui/lei non fosse presente per poterlo fare	6. I have enough confidence in my leader that I would defend and justify his/her decision if he/she were not present to do so
7. La relazione lavorativa con il mio capo è efficace	7. My working relationship with my leader is effective
Servant Leadership	
Il mio capo antepone ciò che è meglio per me a ciò che è meglio per lui/lei	My leader puts my best interests ahead of his/her own
2. Il mio capo fa tutto il possibile per porsi al mio servizio	2. My leader does everything he/she can to serve me
3. Il mio capo sacrifica i suoi interessi per soddisfare le mie necessità	3. My leader sacrifices his/her own interests to meet my needs
4. Il mio capo va ben oltre il suo dovere per soddisfare i miei bisogni	4. My leader goes above and beyond the call of duty to meet my needs
5. Se avessi un trauma personale mi rivolgerei al mio capo	5. My leader is one I would turn to if I had a personal trauma
6. Il mio capo è bravo/a ad aiutarmi con le mie questioni emozionali	6. My leader is good at helping me with my emotional issues
7. Il mio capo ha del talento nell’aiutarmi a riprendermi emotivamente	7. My leader is talented at helping me to heal emotionally
8. Il mio capo è una persona che mi potrebbe aiutare a correggere la mia durezza di sentimenti	8. My leader is one that could help me mend my hard feelings
9. Il mio capo sembra attento/a a ciò che accade	9. My leader seems alert to what’s happening
10. Il mio capo è bravo/a ad anticipare le conseguenze delle decisioni	10. My leader is good at anticipating the consequences of decisions
11. Il mio capo ha grande consapevolezza di ciò che fa	11. My leader has great awareness of what is going on
12. Il mio capo sembra in contatto con quello che succede	12. My leader seems in touch with what’s happening
13. Il mio capo sembra sapere ciò che sta per accadere	13. My leader seems to know what is going to happen
Authentic Leadership	
*Il mio capo*	*My Leader*
Chiede feedback agli altri per migliorare il rapporto con loro	Seeks feedback to improve interactions with others
2. Descrive accuratamente il modo in cui gli altri vedono le sue capacità	2. Accurately describes how others view his or her capabilities
3. Parla seriamente	3. Says exactly what he or she means
4. Ammette i propri errori quando ne compie	4. Is willing to admit mistakes when they are made.
5. Dimostra delle opinioni coerenti con le sue azioni	5. Demonstrates beliefs that are consistent with actions
6. Prende decisioni in base alle sue credenze profonde	6. Makes decisions based on his/her core beliefs
7. Sollecita dei punti di vista che possano sfidare le sue posizioni profondamente radicate	7. Solicits views that challenge his or her deeply held positions
8. Ascolta attentamente diversi punti di vista prima di giungere a conclusioni	8. Listens carefully to different points of view before coming to conclusions
